# History Taking as a Diagnostic Tool in Children With Chronic Cough

**DOI:** 10.3389/fped.2022.850912

**Published:** 2022-04-15

**Authors:** Ahmad Kantar, Julie M. Marchant, Woo-Jung Song, Michael D. Shields, Grigorios Chatziparasidis, Angela Zacharasiewicz, Alexander Moeller, Anne B. Chang

**Affiliations:** ^1^Paediatric Asthma and Cough Centre, Gruppo Ospedaliero San Donato, Bergamo, Italy; ^2^Department of Paediatrics, University Vita Salute San Raffaele, Milano, Italy; ^3^Department of Respiratory and Sleep Medicine, Queensland Children's Hospital, Brisbane, QLD, Australia; ^4^Center for Children's Health Research, Queensland University of Technology, Brisbane, QLD, Australia; ^5^Department of Allergy and Clinical Immunology, Asan Medical Center, University of Ulsan College of Medicine, Seoul, South Korea; ^6^Medicine, Dentistry and Biomedical Science, Queen's University Belfast, Belfast, United Kingdom; ^7^Royal Belfast Hospital for Sick Children, Belfast, United Kingdom; ^8^Primary Cilia Dyskinesia Unit, School of Medicine, University of Thessaly, Larisa, Greece; ^9^Department of Pediatrics, Adolescent Medicine, Teaching Hospital of the University of Vienna, Wilhelminen Hospital, Klinikum Ottakring, Vienna, Austria; ^10^Division of Respiratory Medicine and Childhood Research Center, University Children's Hospital Zurich, Zurich, Switzerland; ^11^Child Health Division, Menzies School of Health Research, Darwin, NT, Australia

**Keywords:** chronic cough, children, history taking, diagnosis, red flags

## Abstract

Chronic cough is a common symptom of many underlying respiratory and non-respiratory disorders and may be associated with less serious causes, such as gastroesophageal reflux and nasal diseases. Chronic cough in children differs from that in adults with respect to its etiologies and management since it can indicate a symptom of an underlying disease in children. Guidelines for managing chronic cough in children are based on recording the history, followed by physical examination, chest radiography, and spirometry. Thus, taking accurate respiratory history for coughing helps delineate the pathophysiological basis of the cause of chronic cough. Detailed history taking enhances the evaluation and treatment, and facilitates a tailored diagnostic identification of likely diagnoses. While studies have described evidence-based red flags in children with chronic cough, the value of skilled physicians regarding history taking has received less attention for the best patient care. In the present article, we outline the major questions comprising a detailed history taking for chronic cough in children.

## Introduction

Coughing in children is one of the most common symptoms primarily resulting from respiratory tract disorders, but may also be associated with a variety of extra-pulmonary causes. Since the underlying pathology can indicate either a benign or more severe condition, an accurate and efficient diagnosis is required to identify effective treatments. Thus, guidelines for evaluating chronic cough in children have been developed to help healthcare practitioners. These guidelines have proven efficacious in improving the quality of life and achieving cough resolution earlier (compared to that in controls) in children presenting to specialist clinics (1) and in those presenting with acute cough who then developed chronic cough (2). These guidelines consist of a thorough history, physical examination, chest radiography, and spirometry (3). History-taking is justified based on its high diagnostic yield, which guides subsequent diagnostic or therapeutic approaches (4, 5).

Over the centuries, knowledge of the anatomy and mechanisms underlying symptoms and signs has been gathered and related to the observed patterns of classical respiratory illnesses. This has made history and examination central and most important in determining a diagnosis and identifying correct investigations and treatment (6). Surprisingly, no studies addressed how medical history data aids chronic cough evaluation in children. While few studies have identified evidence-based red flags or pointers in children with chronic cough, the diagnostic value of skilled history taking has received less attention. Poor knowledge of the key diagnostic features of patient history may lead to inappropriate investigations, resulting in a delay in the diagnosis and management of the disease. Accurate history can efficiently aid clinicians in diagnosing and treating children with chronic cough. The present review aimed to explore the role of expert history taking and evidence-based red flags in diagnosing chronic cough in children and adolescents. The majority of the data presented refer to children/adolescents aged ≤ 14 years (hereafter referred to as “children”).

## The Art of History-Taking

In children, chronic cough is the symptom of an underlying disease (3), and accurate history taking helps identify the cause of the cough as a complete history has a much higher diagnostic yield than that of conventional testing. The art of history taking requires a step-by-step approach, focusing on details and discrepancies, following a thorough review of the history. Keys to the successful history taking of chronic cough include [a] being meticulous and taking sufficient time with the patient, [b] having a calm relaxed setting, and [c] listening carefully to both the patient and family members. Clinicians must have excellent communication skills to gather patient stories, be objective, unprejudiced, and empathic (7). Racial and ethnic minorities report less partnership with physicians, less participation in medical decisions, and lower levels of satisfaction with care. Knowledge of cultural beliefs, behaviors about health and wellbeing and practices of different non-majority groups improve the patient-provider interaction. Current cough guidelines promote specific pointers to direct clinicians to initiate a workup plan often according to the limb of the algorithm (8–10). Moreover, a warning red flag alerts the clinician regarding the presence of a potentially serious problem or those requiring specific immediate action. Clinical years of experience has formed the basis of many specific pointers and red flag alerts in chronic cough, many of which can be identified during history taking, and are of great importance in the diagnosis of children with cough. Recent evidence-based studies have determined the sensitivity, specificity, and likelihood ratios of specific cough pointers and red flag alerts (Table 1).

**Table 1:** Utility of specific cough pointers in differentiating specific coughs from non-specific coughs in children with chronic cough.

**Pointer**	**Reference**	**Design**	**Setting**	**Population**	**Reference standard**	**Sensitivity**	**Specificity**	**PPV**	**NPV**	**PLR**	**NLR**
Wet cough	Marchant et al. (8)	Prospective cohort	Single tertiary hospital in Australia	100 children with CC without known lung or other serious medical conditions (median age 2.8 years)	Specific cough (all causes)	96%	26%	74%	73%	1.29	NR
	Chang et al. (9)	Prospective cohort	Multi-centers in Australia	326 children with CC without a previous diagnosis confirmed by objective tests (asthma, CF, or BE) (mean age 3.3 years)	Specific cough (all causes)	65% (60–71%)	98% (85–100%)	99% (97–100%)	28% (21–37%)	26.15 (3.77–181.48)	0.36 (0.30–0.42)
Wet cough not resolved after 4 weeks	Chang et al. (9)	as above	as above	as above	Specific cough (all causes)	3% (2–7%)	100% (89–100%)	100% (65–100%)	13% (9–16%)	Infinity	0.97 (0.94–0.99)
Wheeze or reversible airway obstruction	Chang et al. (9)	as above	as above	as above	Specific cough (all causes)	21 % (17–26%)	100% (89–100%)	100% (92–100%)	15% (11–20%)	Infinity	0.79 (0.74–0.84)
Exertional dyspnea	Marchant et al. (8)	as above	as above	as above	Specific cough (all causes)	38%	65%	70%	32%	1.06	NR
	Chang et al. (9)	as above	as above	as above	Specific cough (all causes)	3% (2–6%)	100% (90–100%)	100% (63–100%)	10% (10–20%)	Infinity	1.0 (0.9– 1.0)
Chronic dyspnea	Marchant et al. (8)	as above	as above	as above	Specific cough (all causes)	7%	97%	83%	32%	2.25	NR
Recurrent pneumonia	Marchant et al. (8)	as above	as above	as above	Specific cough (all causes)	7%	94%	71%	31%	1.12	NR
	Chang et al. (9)	as above	as above	as above	Specific cough (all causes)	3% (2–7%)	100% (89–100%)	100% (66–100%)	12% (9–17%)	Infinity	0.96 (0.94–0.99)
Hemoptysis	Marchant et al. (8)	as above	as above	as above	Specific cough (all causes)	7%	97%	83%	32%	2.25	NR
Cough associated swallowing	Marchant et al. (8)	as above	as above	as above	Specific cough (all causes)	25%	71%	65%	30%	0.85	NR
Failure to thrive	Chang et al. (9)	as above	as above	as above	Specific cough (all causes)	0% (0–2%)	100% (89–100%)	100% (5–100%	13% (9–17%)	Infinity	1.0 (0.99–1.00)
Feeding difficulties	Chang et al. (9)	as above	as above	as above	Specific cough (all causes)	6% (3–9%)	100% (89–100%)	100% (76–100%)	13% (9–17%)	Infinity	0.94 (0.92–0.97)
Chest pain	Chang et al. (9)	as above	as above	as above	Specific cough (all causes)	0% (0–2%)	100% (89–100%)	100% (5–100%)	13% (9–17%)	Infinity	1.0 (0.99–1.00)
Any cough pointer	Chang et al. (9)	as above	as above	as above	Specific cough (all causes)	100% (98–100%)	95% (82–99%)	99% (97–100%)	100% (89–100%)	20 (5.18–77.21)	0 (0– 0.03)

*CC, chronic cough; PPV, positive predictive value; NPV, negative predictive value; PLR, positive likelihood ratio; NLR, negative likelihood ratio; NR, not reported; FBA, foreign body aspiration*.

## Aetiological Causes of Chronic Cough

In the last 15 years, researchers have reconsidered the aetiology of chronic cough in children. Grouping cough into distinctive classes based on pathophysiology is vital to facilitate a diagnostic approach (11). We present a grouping of nine pathological classes that encompass the most prevalent causes of chronic cough (Table 2). These classes can present as a single aetiology of cough or combinations of more than one such as airway infection due to airway aspiration.

**Table 2:** Major aetiological causes of chronic cough in children^*^ and examples.

1	**Post-infectious**: typically shows spontaneous resolution over time
2	**Airway infections (protracted/recurrent/persistent)**: Protracted bacterial bronchitis, chronic suppurative lung disease, bronchiectasis, cystic fibrosis, immune deficiency/ciliary dyskinesia, alpha-1 antitrypsin deficiency, other chronic infections e.g., tuberculosis and atypical mycobacteria
3	**Airway anomaly**: Primary or secondary tracheobronchomalacia, congenital airway and pulmonary malformation
4	**Airway inflammation**: Asthma, eosinophilic bronchitis, environmental pollutants
5	**Airway aspiration**: Primary airway aspiration, secondary aspiration owing to gastroesophageal reflux, foreign body aspiration
6	**Upper airway associations**: Rhinitis, sinusitis
7	**Tic and somatic syndrome**
8	**Extra-pulmonary**: Drug-induced, cardiac, vagal nerve branches stimulation (e.g., Arnold's ear reflex)
9	**Other specific diseases associated with chronic cough**: Interstitial lung disease or tumors

^*^Some overlap, for example, chronic cough related to primary ciliary dyskinesia can be both infection and inflammation.

Childhood post-infectious cough (typically with natural resolution over time) is a common aetiology in various age groups (12). After viral or bacterial infection of the airway, cough reflex hypersensitivity may continue for weeks (13, 14). In this category, the cough is dry, with no other symptoms (9). This entity belongs to the non-specific cough classification, where watchful waiting is the recommended approach (9, 10). Many cases of post-infection are likely associated with prolonged cough hypersensitivity that takes time to resolve (15). Cough was reported among the most common respiratory symptoms in children 3.2 ± 1.5 months after a SARS-CoV-2 infection (16).

Protracted, recurrent, or persistent airway infections include protracted bacterial bronchitis (PBB), chronic suppurative lung disease, bronchiectasis, cystic fibrosis, immune deficiency, ciliary dyskinesia, alpha-1 antitrypsin deficiency, and tuberculosis. The list is not exhaustive, as any untreated pulmonary infection can cause a chronic cough.

Airway anomalies include primary and secondary tracheobronchomalacia or other congenital airway malformations associated with various respiratory symptoms, such as chronic cough (17). These airway anomalies seem to be predisposed to and are closely associated with chronic recurrent airway infection and consequent inflammation. For example, a recent case-control study demonstrated that the presence of tracheomalacia (defined by European Respiratory Society as >50% expiratory reduction in the cross-sectional luminal area seen on flexible bronchoscopy) is an independent risk factor for bronchiectasis with an adjusted odds ratio of 24.4, and a 95% confidence interval (CI) of 3.4 to infinity (18).

Airway inflammation, such as various asthma phenotypes and eosinophilic inflammation in other airway infections, represent a common aetiology in both children and adults. Typically, mediators released during inflammation in allergic airway diseases can alter the function of the sensory and parasympathetic nervous systems, innervating the airways (19). The effect of inflammation on cough neural processing occurs at multiple peripheral and central sites within the nervous system (20). Moreover, allergen-induced bronchoconstriction and airway eosinophilia are associated with increased cough reflex sensitivity to capsaicin (21).

The airway aspiration class covers primary (during swallowing) and secondary (related to gastroesophageal reflux) airway aspiration and undiagnosed or retained foreign body aspiration (22–24).

Somatic or tic cough is not an uncommon cause of chronic cough in children. Tics usually develop before 10 years of age and exhibit a waxing and waning course, but may increase as the age advances. Tics are prevalent in approximately 1% of children and adolescents (25).

Extra-pulmonary causes of cough include the use of angiotensin-converting enzyme (ACE) inhibitors or conditions that promote stimulation of the vagal branches. Arnold's nerve ear wax, cholesteatoma, or foreign bodies have are reported causes of chronic cough in children and adults (26, 27). Although rare, coughs induced by cardiac pathologies, mostly arrhythmias, have been reported in adults but not in children (28, 29).

Other specific cough aetiology includes specific types of cough, which are not yet diagnosed but correlate with interstitial lung diseases or tumors (30). In this class, numerous heterogeneous signs and symptoms have been reported. As cough can be a common symptom of airway and parenchymal abnormalities, it is not possible to list all of the causes in this paper.

## The Chronic Cough History

Identification of symptoms and signs is the first goal of history taking to establish if specific pointers can help the clinician determine the etiological classification the patient most likely fits, and the algorithm for the treatment most appropriate to follow (10). A structured cough history should be conducted, which includes the mode of onset, severity, cough characteristics, time course/trajectory, and effects of previous treatment. The next stage is to identify the associated respiratory symptoms (breathlessness, wheezing/stridor/snoring, chest pains, haemoptysis) and other extrapulmonary symptoms (e.g., gastroesophageal reflux symptoms). The social context of the child with chronic cough is explored based on the child's age, birth history, and family history, which also includes the impact of cough on children and their families.

## Key Features of the Chronic Cough History

### Age of Onset

A key element of chronic cough history in children is the onset of the cough.

#### Neonatal Onset

Chronic cough that started in and has continued since the neonatal period suggests that specific conditions need to be identified. These include (1) dysfunctional swallowing, (2) airway anomalies (e.g., laryngeal cleft, tracheoesophageal fistula), or (3) primary ciliary dyskinesia. Furthermore, in the context of the management of prematurity, injury to the lung by oxygen toxicity, mechanical ventilation, or infections increases the risk of long-lasting pulmonary impairment. Therefore, clinicians must explore neonatal respiratory distress syndrome, meconium aspiration, neonatal pneumonia, bronchopulmonary dysplasia, and treatment modalities, as well as corticosteroids, surfactants, and advanced respiratory care. Furthermore, congenital cardiac abnormalities, diaphragmatic hernia, tracheoesophageal fistula, or esophageal atresia are associated with long-term sequelae, such as tracheobronchomalacia or bronchiectasis (17, 31).

#### Pre-school Children

Common causes of cough in the preschool age are post-infectious airway infections, airway anomalies, or asthma (32). However, PBB is more common in preschool-aged children and marginally more common in males (33, 34). A study (35) that recruited 903 children presenting with acute cough and followed-up for development of chronic cough found that the risk factors for PBB were: childcare attendance [adjusted relative risk (aRR) = 2.32, 95% CI 1.48–3.63], prior history of chronic cough (aRR = 2.63, 95% CI 1.72–4.01), and age <2-years (<12-months: aRR = 4.31, 95% CI 1.42–13.10; 12- <24 months: aRR = 2.00, 95% CI 1.35–2.96). Factors that decreased the risk were baseline diagnoses of asthma/reactive airway disease (aRR = 0.30, 95% CI 0.26–0.35) or bronchiolitis (aRR = 0.15, 95% CI 0.06–0.38) (35).

#### School Children and Adolescents

The causes of chronic cough among older children and adolescents become more similar to those of adults with asthma, upper airway associations, and gastroesophageal reflux becoming more prominent (11). Additionally, cough can result from chronic suppurative lung disease and bronchiectasis in children who suffered recurrent lower respiratory infections during early childhood.

### Mode of Onset

#### Abrupt Onset

Determining the onset of cough is vital for all children, regardless of how long they have been coughing (Table 3). This is crucial to rule out FB inhalation. Retained inhaled FB is common in young children between 0 and 3 years, and this may be unrecognized because a detailed history of the mode of onset was not explored. It is important to remember that the choking/spluttering episode may not have been observed by parents, or the inhalation event may not cause marked symptoms. The abrupt onset of coughing in a healthy child is thus a red flag that alerts the clinician about the possibility of an inhaled FB. The key clinical diagnostic feature is penetration syndrome, corresponding to respiratory defense reflexes (expulsive cough and laryngeal spasm) in response to a FB. There may also be asphyxia elements, such as cyanosis associated with coughing (36). Symptoms vary according to FB site in the airways. When the FB is trapped in the larynx or trachea diagnosis is immediately suggested owing to respiratory distress or stridor. In comparison, a positive diagnosis of FB bronchial may be challenging when few or no symptoms are identified. Kiyan et al. calculated the sensitivities, specificities, and positive and negative predictive values of clinical history, symptoms, physical examination findings, and radiological findings in patients with suspected FB aspiration (37). The sensitivity and specificity of the clinical history were 90.5 and 24.1%, respectively. Moreover, the sensitivity and specificity of symptoms reported were 97.8 and 7.4%, physical examination findings were 96.4% and 46.3%, and radiological findings were 71.7 and 74.1%, respectively (37). The outcomes of the literature review of 12,979 cases revealed that most patients with aspirated FB are children younger than 3 years of age (38). A history of abrupt cough is highly sensitive to FB aspiration (varied from 41 to 93.4%), but not specific, with reported specificity ranging from 8.3 to 55.3%) (38). However, a history of cyanosis (98.1–100%) or stridor (65.5–100%) at the onset is very specific to FB but not very sensitive (38).

**Table 3:** Mode of onset and potential diagnostic category.

**Mode of onset**		**Diagnostic category**
Abrupt 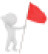		Airway foreign
		body aspiration
Gradual	Progressing	All causes
	Stuttering	

#### Gradual Onset

If the onset of cough is progressive or stuttering, it will be difficult to attribute it to a specific category. If parents could accurately recollect the history, a child with a runny nose when the cough started may signify an upper respiratory tract infection, with the most likely cause being an airway infection resulting in a post-infectious cough.

### Cough Trajectory

#### Continuous (or Static but On-Going) Chronic Cough

Children with chronic cough present with cough daily, but the cough may worsen when there is a new respiratory tract infection. The cause of this chronic cough can fall into any diagnostic category (Table 4).

**Table 4:** Cough trajectory and potential diagnostic category.

**Cough trajectory**	**Diagnostic category**
Continuous	All causes
Subsiding	Post-infectious
Recurrent acute cough	Recurrent respiratory infections, all causes
Relentlessly progressive or	Airway infection
static but on-going	Airway anomaly
	Airway aspiration
	Other specific diseases

#### Recurrent Acute Cough

Chronic cough occurs when coughing is continuous and unceasing. Many children frequently experience recurrent coughing when they have upper respiratory tract infections. Children aged 2–5 years may experience several episodes of respiratory tract infections annually, especially if they attend day-care (39). Distinguishing recurrent acute cough due to recurrent infections is vital to historical details, assisting in delineating a chronic cough history. Further, in many respiratory infections, cough is often the last symptom that disappears. Problem coughing with each respiratory viral infection with only a short period of resolution may blend into the next infection and can be reported erroneously as chronic cough (40).

A careful history and enquiry on the timing of coughing can help in the differential diagnosis, asking for the parents to recall “when the child had days free of cough?” This is related to the onset of new upper respiratory tract infection symptoms, such as rhinorrhoea. The observation of coughing after the child's withdrawal from day-care or holidays often confirms the diagnosis.

#### Subsiding Cough

Many children with prolonged coughing (for longer than 4 weeks), after an upper respiratory tract infection, suffer from what is identified as a post-infectious cough. When the trajectory course of coughing suggests that it is waning and no other signs and symptoms are present, further observation should be made to ensure that the cough resolves completely.

#### Relentlessly Progressive Coughing

Prolonged coughing, which progressively worsens, requires further investigation and management. Potential causes vary and may include: (a) a retained FB, (b) *Bordetella pertussis* infection, (c) an expanding airway compressive lesion, for example, malignancy, and d] progressive airway infection (e.g., mycobacteria or fungi).

### Type of Cough

Coughing is a sudden expulsion of air from the airways, which is characterized by a typical sound. The sound of a cough is associated with the vibration of larger airways and laryngeal structures during turbulent flow during expiration (41). This sound is specific and helps to identify cough, which is distinct from other vocal manifestations. The coughing sound is an important symptom, which is different from hundreds of diseases. Changes in its characteristics may have considerable value in identifying the mechanisms of airway pathology in respiratory diseases.

#### Cough Sounds

The cough sound provides information about the pathophysiological mechanisms of coughing by indicating the structural nature of the tissue that leads to certain patterns of cough. Under certain pathological conditions, cough sounds can help in the diagnosis (Table 5). For example, barking/brassy seal-like cough sounds are indicative of airway anomalies, such as tracheobronchomalacia or somatic cough. In many tracheobronchomalacia cases, parents report that they can identify the type of cough by hearing their children's coughing. Paroxysmal spasms of severe cough followed by an inspiratory whooping sound can be characteristic of pertussis. Barking honking cough is typical of somatic cough syndrome and tic cough. In infants, a staccato cough is indicative of chlamydia infection.

**Table 5:** Cough sound and potential diagnostic category.

**Cough sound**	**Diagnostic category**
Barking/brassy seal-like	Airway anomaly
	Tic and somatic syndrome
Whooping/Paroxysmal/spasmodic	Post-infectious (pertussis)
	Other specific diseases
Staccato	Post infectious (Chlamydia)
	Other specific diseases
Honking	Tic and somatic syndrome

#### Cough Quality – Wet or Dry?

An essential component of a cough history includes determining if the cough is wet/productive or dry, which is vital information concerning the pathology of the disease (Table 6). When the cough is mixed (sometimes dry and sometimes wet), it is considered a wet cough.

**Table 6:** Type of cough and potential diagnostic category.

**Type of cough**	**Diagnostic category**
Dry	Post-infectious
	Airway inflammation
	Tic and somatic syndrome
	Extra-pulmonary
	Other specific diseases (e.g., tumors)
	Upper airway associations
Wet 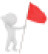	Airway infection
	Airway aspiration
	Airway anomaly
	Upper airway associations
	Other specific diseases

Cough is a vital mechanism for removing mucus from the airways. Cough sound is effective in detecting mucus in the larger airways, as opposed to the smaller airways, because the rheological properties of mucus influence cough sounds. Additionally, shear stress through mucus secretions from the airways contributes to these sounds. In healthy adults, the area occupied by the mucus gland constitutes approximately 12% of the bronchial wall. However, in children, it is approximately 17% of the bronchial wall (42), leading to greater mucus secretion during childhood. The difference in composition suggests that mucus gland hypertrophy is more significant in children than in adults. After the accumulation of mucus in the lung, clearance of mucus by the high-velocity airflow associated with cough often becomes the sole mechanism for mucus clearance (42). In a normal cough, the high airflow velocity creates high shear stress, which clears the foreign matter and secretions off the bronchial wall, propelling them toward the larger airways and trachea.

Cough constitutes an important backup mechanism to the mucociliary escalator, which has been the primary mechanism to remove mucus from the lungs of patients with lung disease. A cough initiated deeply from the lungs is associated with an initial deep inspiration that allows air to get behind secretions within the distal airway. However, cough initiated from the upper larynx is not associated with an initial deep inspiration.

If a child's cough is wet, a phlegmy, rattly sound with the cough is emitted, suggesting the presence of secretions in the airways. Airway secretions are always present in wet cough. Moreover, wet cough in children, as determined by clinicians and parents, has good clinical validity (41). However, clinicians should interpret parental reports of a child's cough with some caution, in that one person's “dry” cough may very well be another's “wet” cough. Indeed, Morey et al. observed the unreliability of a 24 h history of reported cough quality (wet/dry) by carers of indigenous children compared with objectively recorded cough (43). Hence, clinicians should endeavor to hear the cough themselves either during the consultation or ask the parents to record it (44). Smart mobile phones are increasingly being employed to record cough, which greatly helps physicians identify the type of cough sound.

Wet cough is associated with increased airway infections, airway anomalies, airway aspiration, and other less common specific diseases. Conversely, dry cough is associated with post-infectious conditions, tic and somatic syndrome, extra-pulmonary cough, or other less common specific diseases. In some cases, cough sounds may alternate between dry and wet; in this case, the cough is considered to be wet. Chang et al. argued that wet cough is categorized as a specific cough (those that require treatment) and non-specific cough (likely to resolve without treatment) (9). Wet cough has a positive likelihood ratio (LR) of 26.2 (95%CI 3.8–181.5) (9). Although the absence of other pointers (associated signs and symptoms of coughing illness discussed below) did not significantly change the pre-test probability (negative LR close to 1). The absence of all pointers (including wet cough) had a strongly negative LR of 0 (95% CI, 0–0.03) (Table 1). Hence, chronic dry cough without any cough-specific pointers in children, based on the outcome of normal chest radiographs, can be safely managed using the watchful waiting approach.

### Presence of Expectorated Sputum

Some children (mainly older children) can expectorate or cough up sputum, and questioning about the properties of the mucous/sputum should be included (Table 7). Mucous within the airways can be associated with airway inflammation or infection, which can be eosinophilic or neutrophilic, both, or lymphocytic. Identification is vital during the diagnosis (45). In adults, purulent sputum, a yellow to green color, is associated with neutrophilia and possible bacterial infections (46, 47). In children, the color (and amount) of airway secretions observed during bronchoscopy is associated with bacterial infection (48). Therefore, bacteriological testing is recommended. A purulent expectorate can be associated with airway infection, airway aspiration, or other specific diseases, and in some cases, airway anomalies.

**Table 7:** Expectorate and potential diagnostic category.

**Characteristics of**	
**the expectorated sputum**	**Diagnostic category**
Absent	No specific indication
Clear	Airway aspiration
	Upper airway associations
	Other specific diseases
	Non-infective airway inflammatory process
Purulent	Airway infection
	Airway aspiration
	Chronic suppurative airway disease
	Airway anomaly
Haematic	Airway infection
(haemoptysis) 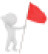	Bronchiectasis
	Arterio-venous malformation
	Other specific diseases (e.g., hereditary
	haemorrhagic telangiectasia)
Casts	Other specific diseases (plastic bronchitis)

Collecting sputum specimens is not feasible in small children, and the risk of obtaining low-quality sputum culture is high; however, these can be obtained in older children (49). Induced sputum may be feasible for young children. A clear expectorate indicates excess secretions that may be related to airway aspiration or upper airway disease.

True haemoptysis is a characteristic of severe underlying conditions. Examples include airway infection (e.g., undiagnosed tuberculosis), bronchiectasis, and other specific diseases (e.g., arteriovenous malformation, tumor). It is important to remember that a child spitting out some blood with a cough is not necessarily true haemoptysis–blood can originate from the throat or indeed from cheek biting.

The cough with expectoration of branching airway casts is characterized by plastic bronchitis. Pediatric cardiothoracic surgeries, infections, and inflammatory processes are among the conditions associated with cast formation (50).

### Triggers of Cough

Events that immediately precede and seem to trigger a cough or worsen cough should be recorded, which can help arriving at a specific diagnosis for the cough (Table 8). Parents and older children should be asked if they can identify symptoms that worsen the cough, such as exercise in cold air, changes in season, meals/feeding, or lying down or body position. Cough can be triggered at the time of meals and feeding points, leading to aspiration syndrome. Cough triggered by physical activity is typically caused by airway inflammation and associated hyperreactivity (asthma). Cough triggered by an allergen is often caused by airway inflammation or upper airway associations. Cough triggered by a change in body position can be caused by airway aspiration, airway anomalies, or other specific diseases. Stress can trigger cough in children with motor or phonic tics or Tourette syndrome.

**Table 8:** Triggers of cough and potential diagnostic category.

**Trigger**	**Diagnostic category**
Physical activity	Any cause
	Airway hyper-reactivity/asthma phenotype
	Eosinophilic airway inflammation
	Upper airway associations
Feeding/meals 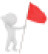	Airway aspiration
	Airway anomaly (e.g., tracheo-esophageal
	fistula)
Allergens	Upper airway associations
	Airway inflammation
Pollution (indoor or outdoor)	Upper airway associations
	Airway inflammation
	Post-infectious
Tobacco smoke and e-cigarettes	Upper airway associations
	Airway inflammation
	Post-infectious
Fog	Upper airway associations
	Airway inflammation
	Post-infectious
Body position 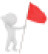	Airway anomaly
	Airway aspiration
Stress	Tic and somatic syndrome
Temperature (cold)	Airway hyper-reactivity/asthma phenotype

Environmental triggers can exacerbate cough and must be addressed by clinicians. For example, a history of exposure to tobacco, e-cigarettes, and environmental smoke (51, 52) can trigger a cough. Parental reporting of cough is less accurate if parents are smokers (53), which may be the reason behind the child's continuous cough. In many developing countries, indoor cooking and heating may contribute to the development of lung disease. Hobbies, such keeping or working with birds, may present a risk of pulmonary infection (54).

### Variability Pattern of Cough Over Day and Night

A history of the variability of cough during the day and night can provide clinicians with important information about the cough and guide them toward a likely diagnosis (Table 9). An exclusively dry diurnal cough in the absence of red flags and normal chest radiography can signal somatic cough, while the presence of night-time cough rules out a diagnosis of somatic cough (10). A pre-dominantly morning wet cough is highly suggestive of chronic airway infections, such as bronchiectasis. A pre-dominant night cough is often attributed to airway aspiration or airway hyper-reactivity/eosinophilic airway inflammation related to asthma. However, clinicians should be aware that nocturnal cough is often inaccurately reported when compared to objective recordings (55, 56).

**Table 9:** Variability during the day and potential diagnostic category.

**Variability**		
**pattern**		**Diagnostic category**
Only diurnal		Tic and somatic syndrome
Pre-dominantly	Uniformly throughout day	All causes
diurnal	Mostly morning	Airway infection/Bronchiectasis
		Airway aspiration
Pre-dominantly		Airway aspiration
nocturnal		Airway inflammation–asthma
		Other specific diseases
Diurnal and		All causes
nocturnal		

### Response to Prior Cough Treatments

Evaluating the response to pharmacological and non-pharmacological interventions before treatment may help identify the cause of cough. The medication type, dosage, duration, and method of delivery are all important factors when discussing a response to prior cough treatments (Table 10). For example, when assessing response to inhaled corticosteroids, the method of delivery, technique of inhalation, dose, frequency, and duration of the trial should be assessed as a short duration (e.g., 3 days) or suboptimal delivery represents an inadequate treatment trial.

**Table 10:** Prior therapeutic intervention.

**Prior therapeutic interventions (drug/dosage/duration/delivery** **method/response)**
Antibiotics: type, dosage and duration
Oral or inhaled corticosteroids
Bronchodilators
Anti-gastroesophageal reflux drugs
Anti-histamines
Mucoactive drugs
Narcotics
Cough suppressants

An appropriate medication trial over a certain period may help to exclude diagnosis and reduce the scope of the investigation. An absence of response to an appropriate antibiotic for an appropriate period with the correct dose (typically an appropriate dose for a minimum of 2 weeks or 4 weeks) suggests that the diagnosis may not be simply PBB (57). Goyal et al. demonstrated that among 105 children with persistent cough, 88 (83.8%) had bronchiectasis despite at least 4 weeks of antibiotic treatment. Of the 24 children whose cough resolved after antibiotic treatment, only six (25.0%) were diagnosed (adjusted OR 20.9; 95% CI 5.36–81.8) (58). The authors concluded that further investigations, including a multi-detector computerized tomography scan, should be considered in a child with a chronic wet cough that persisted after 4 weeks of oral antibiotics. However, reducing the likelihood of underlying bronchiectasis and responding to a single prolonged course of antibiotics does not completely exclude this diagnosis.

Oral or inhaled steroids are effective in treating eosinophilic inflammation (59, 60). Thus, ineffective treatment (assuming it has been correctly delivered) can exclude the presence of eosinophilic inflammation. If narcotics or cough suppressants are used, they must be stopped, and cough should be re-evaluated.

In adult patients with chronic cough, ACE inhibitors are considered wholly or partially the cause (61). Remarkably, the prevalence of ACE inhibitor-induced cough in adults is in the range of 5–35%; in children, it is reported sporadically. Alharbi et al. (62) found that such instances increased with age until a plateau was attained in middle adulthood (40–59 years). The incidence of cough in children receiving ACE inhibitors, as reported by Baker-Smith, was low (3.2%), similar to that in children receiving angiotensin receptor blockers (1.8%) (63).

## Other Key Features of History

### Associated Symptoms and Signs

Apart from cough, questions on any associated symptoms and signs form part of the history taking as it can help to determine the cause of chronic cough and/or the need to undertake further investigations (10). Their presence or absence provides key elements for establishing a diagnostic algorithm (Table 11). Most of these associated symptoms are considered to be red flags. Dyspnoea, chest pain, cyanosis, haemoptysis, haematemesis, fever, and apnoea are red flags to airway infection, airway anomalies, airway aspiration, or other specific diseases. Choking, regurgitation, spitting, vomiting, epigastric pain, and heart pain indicate aspiration syndrome. Neck posturing may indicate an airway anomaly or aspiration syndrome. Wheezing is indicative of airway inflammation (i.e., asthma), airway anomaly (i.e., tracheomalacia), or other specific diseases (9).

**Table 11:** Associated symptoms and potential diagnostic category.

**Associated symptoms**	**Diagnostic categories**
Dyspnoea (at rest or	Airway anomaly
exertional) 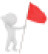	Airway inflammation
	Airway infection
	Other specific diseases (any pulmonary cause)
Chest pain 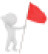	Airway anomaly
	Airway inflammation
	Airway aspiration
	Other specific diseases
Cyanosis 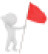	Airway anomaly
	Airway inflammation
	Airway aspiration
	Other specific diseases
Stridor 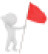	Airway anomaly
	Other specific diseases (e.g., laryngeal abnormality)
Fever 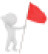	Airway infection
	Other specific diseases
Regurgitation/Spitting/ vomiting 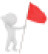	Airway aspiration
Choking during feeding^*^	Airway aspiration Other specific diseases
Haematemesis 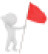	Airway infection
	Other specific diseases
Haemoptysis 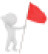	Airway infection
	Other specific diseases
Apnoea 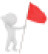	Airway anomaly
	Airway aspiration
	Other specific diseases
Wheezing	Airway anomaly
	Airway inflammation
	Airway infection
	Other specific diseases
Hoarseness	Airway aspiration
	Other specific diseases (e.g., laryngeal abnormality)
Epigastric pain	Airway aspiration
	Other specific diseases
Heartburn	Airway aspiration
	Other specific diseases
Neck posturing (dystonic,	Airway aspiration
spontaneous	Airway anomaly
hyperextension)	Other specific diseases

### Concomitant Disease Conditions

The presence of a disease may be the underlying cause of chronic cough, and its investigation is vital (Table 12). Moreover, previous hospitalisations or treatment for pulmonary diseases should be questioned. Congenital anomalies of the aero-digestive tract after surgical intervention, cardiac anomalies, and congenital syndromes are associated with airway infections, airway anomalies, or other specific diseases. Neurodevelopmental anomalies are frequently associated with airway aspiration or other diseases. Diagnosed immunodeficiency, primary or secondary airway anomalies, airway aspiration, or other specific diseases can cause airway infections and chronic cough. Moreover, rhinitis, sinusitis, and allergic diseases are upper airway coughs.

**Table 12:** Concomitant disease conditions and potential diagnostic categories.

**Concomitant disease conditions**	**Diagnostic categories**
Congenital anomalies of the aero-digestive	Airway aspiration
tract	Airway anomaly
	Other specific diseases
Cardiac abnormalities	Airway anomaly
	Other specific diseases
Neurodevelopmental abnormalities	Airway aspiration
	Airway anomaly
	Other specific diseases
Immunodeficiency/ Immunosuppression	Airway infection
conditions	
Congenital syndromes	Airway anomaly
	Other specific diseases
Weight loss	Airway infection
	Other specific diseases
Failure to thrive	Airway infection Airway aspiration
	Other specific diseases
Tumor	Airway anomaly
	Other specific diseases
Chronic rhinitis/sinusitis	Upper airway associations
	Airway inflammation
	Other specific diseases

Tumor and its therapeutic management are associated with cough. The presence of other tic-related symptoms (e.g., excessive blinking of eyes and twirling of hair) should raise the suspicion of a tic-associated cough. Weight loss and failure to thrive are red flags indicating airway infection, airway anomalies, airway aspiration, or other diseases (9).

### Risk Due to Exposure to Infections

Epidemiological factors must be considered when evaluating chronic cough (Table 13). Non-vaccinated children are at a higher risk of developing infectious diseases. Since this is the case with immunization, natural *B. pertussis* infection may fail to permanently protect patients against pertussis (64). Numerous studies have documented that a second episode of pertussis can occur after a few years. Moreover, the emergence of mutated strains *of B. pertussis* can cause re-infection (65). Visiting or living in an endemic area that can increase the risk of contagious diseases, such as tuberculosis, HIV, or parasitic infections, should be investigated.

**Table 13:** Questioning about risks of exposure to infections.

**Risk factors of exposure to infections**
Day-care attendance
Lack of vaccinations
Cough in family members/relatives
Traveling to endemic areas
Epidemiological risk factors e.g., pollution, settings with high TB prevalence

A further factor to consider when discussing chronic cough with parents is the child's exposure to viral infections. Children who attend childcare have a higher risk of recurrent respiratory infections. As mentioned above, among other factors, childcare attendance was an independent risk factor for the diagnosis of PBB (aRR = 2.32, 95% CI 1.48–3.63) (35).

## Family History

Family history provides key clues to the presence of genetic pulmonary diseases, such as cystic fibrosis, alpha 1-antitrypsin deficiency, hereditary haemorrhagic telangiectasia, immotile cilia syndrome, situs inversus, spontaneous pneumothorax, atopy, asthma, and immunodeficiency syndromes (66). Moreover, careful history taking can uncover more common familial diseases. Family history should encompass at least three generations to account for sex-linked traits. Family history can also identify exposure to tuberculosis or other contagious diseases.

## Impact of Cough

The cough history should also include an evaluation of the impact of cough on children and their families. This includes the most troubling aspects (e.g., sleep) and the impact of the cough on the child (e.g., schooling/preschool) and their parents (e.g., work). Understanding these aspects will assist counseling. Validated parent and child chronic cough specific quality of life questionnaires (PC-QoL (67) and CC-QoL (68), respectively) are available. The short-form of PC-QoL (PC-QoL-8), consisting of only eight questions (56), can be used in the clinic setting. Alternatively, a simple visual analog score (1–10) (56) can also be used to assess the impact and response.

## Summary

Effective evaluation of children with chronic cough is vital, and the use of management pathways specific to children (10) is efficacious. Determining the underlying diagnosis is one of the primary goals in the management of chronic cough in children, followed by targeted investigations or, in some cases, treatment trials. This is contrary to what has been proposed by some groups in adults, where chronic cough itself is considered a syndrome.

Based on the existing guidelines on chronic cough in children, the initial step of management consists of history taking followed by physical examination, chest radiography, and spirometry (in older children who are cooperative). Meticulous and thorough history taking is a cornerstone to this process, for which the primary cost is the clinician's time. Acquiring the necessary history requires exhaustive questioning, leaving sufficient time and careful listening. Physicians should assemble diagnostic clues through unhurried history taking to make a presumptive diagnosis. The process is different from on-the-spot rapid diagnosis, a popular “protocol-driven” medicine. A step-by-step approach is needed, paying attention to details and discrepancies by meticulously reviewing history.

After robust history taking, physical examination, chest radiography, and spirometry may confirm or raise diagnostic reliability. Once a diagnostic probability of the cough is undertaken, the next step in the guidelines includes evidence-based treatment pathways that include PBB (57), bronchiectasis (69), airway aspiration (23), somatic and tic cough (70), pertussis (71) or asthma (72). These management pathways guide clinicians in identifying children who require immediate referral to tertiary care or at which stage to refer to others.

The burden of chronic cough in children is high, with over 80% of children having five or more doctor visits within 12 months, and 53% have more than doctor visits within the same period (73). Stress is the primary contributor to parent's emotional distress. A thorough chronic cough history taking may decrease the burden on families by allowing accurate and timely diagnosis in primary care for some children, and more appropriate early referral to tertiary care.

Some limitations were identified in our history-taking approach, which included patients who presented an overlap of symptoms of more than one diagnostic class. Furthermore, the symptoms of a secondary diagnosis, described as a pathological condition whose treatment does not result in the resolution or improvement of the cough, should be carefully considered (74). In these cases, it is critical to differentiate between primary and secondary diagnoses. In a prospective study in children with chronic cough, Marchant et al. (74) reported that in children with a primary diagnosis of PBB, 55% had a secondary diagnosis (e.g., airway malacia and gastroesophageal reflux).

Chronic cough in children remains a diagnostic challenge in clinical practice. Although new diagnostic tools have been introduced in the field of respiratory medicine, allowing the severity, frequency, and timing of cough to be measured objectively using automated cough measurement devices (75, 76). Nevertheless, history taking remains a primary step in diagnosing children with chronic cough. A combination of a thorough history and the use of cough management protocols or algorithms is likely to improve clinical outcomes and decrease the burden on these children and their families.

## Author Contributions

AK, JM, and AC: study conception and design. W-JS: analysis and interpretation of data. AK, JM, MS, GC, AZ, and AM: draft manuscript preparation. All authors reviewed the results and approved the final version of the manuscript.

## Conflict of Interest

The authors declare that the research was conducted in the absence of any commercial or financial relationships that could be construed as a potential conflict of interest.

## Publisher's Note

All claims expressed in this article are solely those of the authors and do not necessarily represent those of their affiliated organizations, or those of the publisher, the editors and the reviewers. Any product that may be evaluated in this article, or claim that may be made by its manufacturer, is not guaranteed or endorsed by the publisher.
